# Comparing forceps and self-assembled intraocular rare earth magnet in removing metallic intraocular foreign bodies in 25-guage vitrectomy

**DOI:** 10.1186/s12886-024-03343-7

**Published:** 2024-02-21

**Authors:** Huajin Li, Kailing Zheng, Huihang Wang, Maosong Xie

**Affiliations:** Department of Ophthalmology, The First Affiliated Hospital of Fujian Medical University, No. 20 Chazhong Road, 350005 Fuzhou, China

**Keywords:** Intraocular foreign body, Intraocular rare earth magnet, Forceps, Pars plana vitrectomy

## Abstract

**Purpose:**

To compare the efficacy and efficiency of self-assembled intraocular rare earth magnet and forceps in removing intraocular foreign bodies(IOFBs) undergoing 25-gauge(G) pars plana vitrectomy.

**Methods:**

A total of 30 patients with metallic IOFB underwent 25-G PPV were enrolled into this study. Self-assembled intraocular rare earth magnet were used in 15 patients(bar group), and forceps were used in 15 patients(forceps group). Success rate of removing IOFB, time taken to remove IOFB, incidence of IOFB slippage and fall, iatrogenic retinal damages were compared between the two groups.

**Results:**

There was no significant difference in success rate of removing IOFBs between the groups(93.3% and 100%, *P* > 0.99). The median time taken of removing FB was significantly shorter in bar group than in forceps group(112 and 295 s, *P* = 0.001). None of the patients in bar group had IOFB slippage and fall, or related iatrogenic retinal damage in the process of removal. In forceps group, IOFB slippage and fall during removal were observed in 7 of 15(47.6%) patients, related iatrogenic retinal injuries were recorded in 6 of 15(40.0%) patients, both were significantly higher than bar group(*P =* 0.003 and *P =* 0.017, respectively).

**Conclusions:**

Compared with forceps, the assembled intraocular magnet can greatly reduce the possibility of IOFB slippage and fall, prevent related iatrogenic retinal damage, and shorten the time taken to remove IOFB. The assembled intraocular magnet can be an useful tool in removing metallic IOFBs in PPV.

## Introduction

Retained intraocular foreign body(IOFB) is an ophthalmic emergency, which account for 14–29% of open-globe injuries [1, 2]. About 78–96% of IOFB are metallic in nature, the majority of which are magnetic [2–4]. Retained metallic IOFBs often cause various damage to ocular structures and serious complications such as metallosis, retinal detachment and endophthalmitis. Thus immediate surgical extraction is required [5].

In the past decades, pars plana vitrectomy(PPV) has been advocated and widely used in removing IOFB [6, 7]. Comparing with external electromagnet, PPV provides a direct visualization and allows a timely management of vitreous hemorrhage and retinal detachment, which would result in better anatomic outcomes, visual prognosis and reduce the risk of endophthalmitis development [8].

Forceps is the most commonly used instrument in the process of removing IOFB in PPV. In clinical practice, vitreoretinal surgeons may experience troubles in grasping a proportion of IOFB using forceps. Difficulties in controlling and grabbing IOFB often lead to slippage and fall. Dropped foreign body may easily cause iatrogenic retinal injuries and even damaged visual function. In this study, we introduced a self-assembled intraocular rare earth magnet, and compared its efficacy and efficiency with common forceps in removing metallic IOFBs in 25-guage vitrectomy.

## Patients and methods

### Subjects

This was a retrospective case-control study. Medical records of all 30 patients with metallic intraocular foreign bodies who underwent 25-gauge(G) PPV at the First Affiliated Hospital of Fujian Medical University between Aug 2019 to Oct 2022 were reviewed. The control group included 15 patients who underwent IOFB removal using intraocular forceps between Aug 2019 to Dec 2020 (forceps group). The study group consisted of 15 patients who underwent IOFB removal using a self-assembled intraocular rare earth magnet between Jan 2021 to Oct 2022 (bar group). This study was approved by the Institutional Review Board of the First Affiliated Hospital of Fujian Medical University and complied with the principles of the Declaration of Helsinki(MRCTA,ECFAH of FMU [2015084-2]). A signed informed consent form was obtained from all participants.

The following data were compared between the two groups: age, sex, foreign body location, mean size(length and width) of the foreign bodies, success rate of removing IOFB, site of removing IOFB, time taken to remove IOFB, IOFB slippage and fall, iatrogenic retinal damage, iatrogenic retinal break and retinal detachment. The time taken to remove IOFB started to be calculated from the moment the forceps or magnetic bar was inserted into vitreous cavity until the moment IOFB was completely removed.

### Assemble of the rare earth magnet

**Fig. 1:**
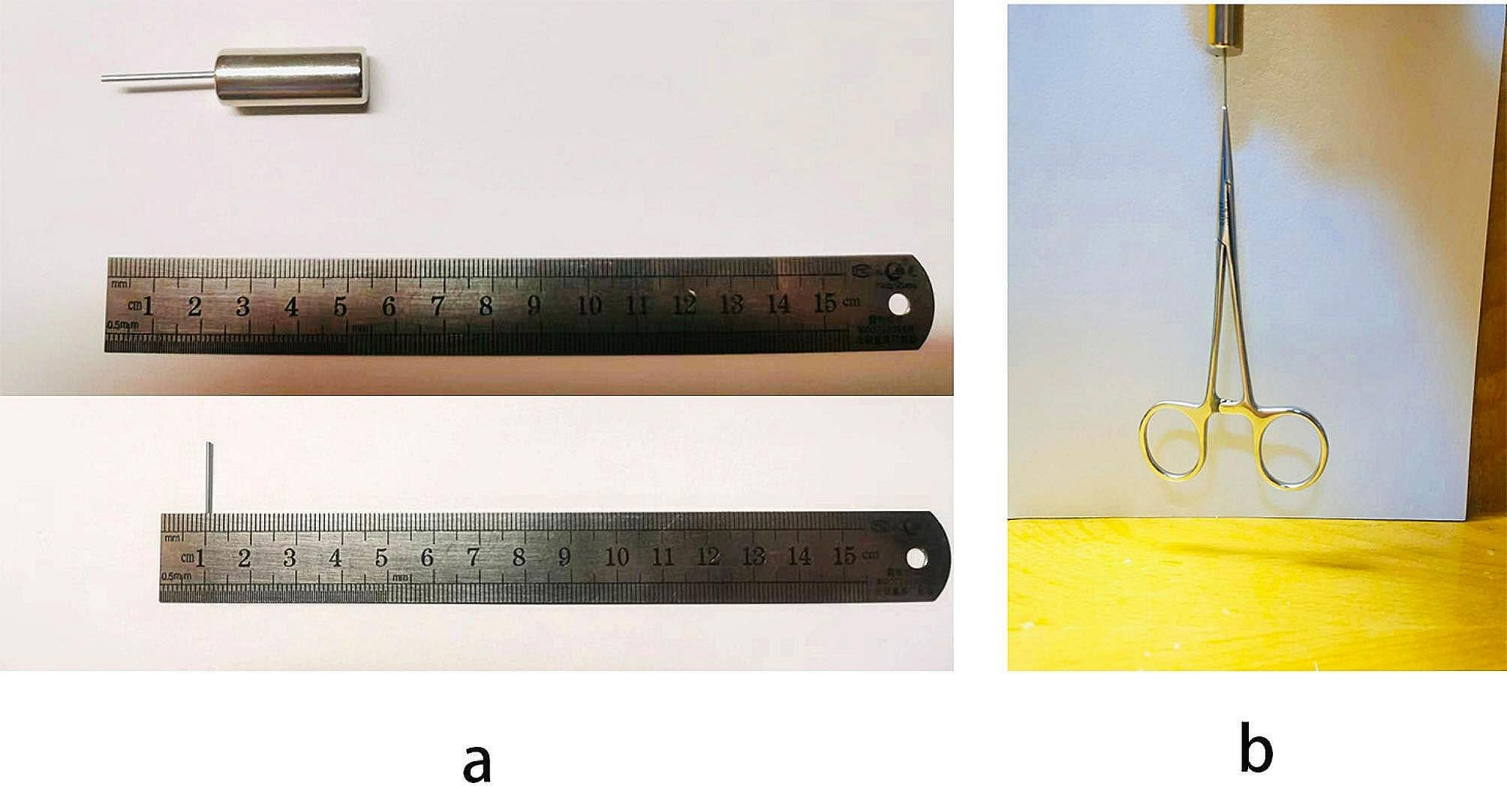
(**a**) The assembled intraocular rare earth magnet consist of a magnetic bar and a cylindrical strong magnet(NdFeB magnet). The magnetic bar is approximate 1.2 mm in diameter and 24 mm in length. (**b**) The assembled intraocular rare earth magnet lifting a vessel clamp

A magnetic bar with approximate 1.2 mm in diameter and 24 mm in length was connected with a cylindrical strong magnet, as shown in Fig. 1a. Both were made of Neodymium-iron rare earth permanent magnet alloy(NdFeB magnet). The assembled rare earth magnet can easily lift a vessel clamp(Fig. 1b). The assembled rare earth magnet was soaked in 5% povidone iodine solution for 1 min, and fully rinsed in sterilized saline solution before use.

### Surgical technique

**Fig. 2:**
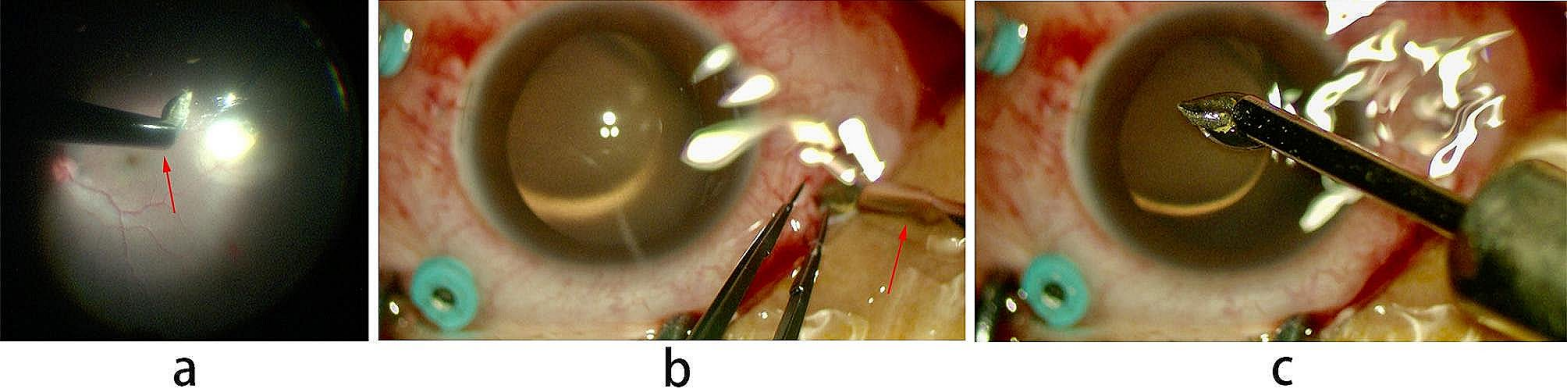
(**a**) The IOFB was attracted and adhered to the intraocular magnetic bar. (**b**) The IOFB was removed through the enlarged scleral incision site. (**c**) The IOFB was completely removed from the eye. The IOFB was approximate 2.5 mm×1.5 mm in size. Red arrow refers to the magnet bar

All surgeries were performed by one surgeon(Maosong Xie) under retrobulbar anesthesia. All operation eyes were fully mydriatic before surgery. Vitrectomy was performed with 25-G instruments and the Constellation Vision system (UHS Alcon Constellation; Alcon Laboratories, Inc, Fort Worth, TX). Resight 700 (Carl Zeiss Meditec AG, Jena, Germany) wide-angle viewing system had been used in all cases during surgery. At the beginning of the surgery, 3 valved 25-G trocar cannulas were inserted at 2 o’clock, 8 o’clock and 10 o’clock position, 3.5 mm posterior cornea limbus. Obvious traumatic cataract was extracted using phacoemulsification technique or vitrectomy probe. After shaving the anterior vitreous, a posterior vitreous detachment was induced when necessary, and a core vitrectomy was performed. The IOFB was identified and dissociated from surrounding tissue. Before removing the IOFB, one of the sclerotomy port was enlarged. For the patients in control group, the IOFB was grabbed with forceps via the enlarged sclerotomy port. For the patients in study group, the IOFB was attracted by the magnetic bar under direct vision (Fig. 2a) and removed via the enlarged sclerotomy port (Fig. 2b-c). A careful check of retina was performed. Retinal lesions or retinal breaks were treated with endo-photocoagulation. Fluid-gas exchange and silicone oil injection were performed in cases with retinal detachment. Intravitreal antibiotics was administrated in all subjects.

### Statistical analysis

Statistical analysis was performed using SPSS software(version 22.0). Shapiro-Wilk test was used to assess the normality of measurement variables. If the measurement variables were normally distributed, the mean and standard deviation was used, while the median and interquartile spacing was used for skewed distributed variables. Categorical variables were described by proportions. Student-t test and Mann-Whitney *U* test were used for the comparison of normally distributed variables and skewed variables, respectively. Chi-square test and Fisher’s exact test were used for quantitative data. *P* values < 0.05 was considered statistically significant.

## Results

The average age was 32.5 ± 8.2 years(range, 21–49 years) in forceps group, and 36.2 ± 11.5 years(range 22–58 years) in bar group (*P* = 0.901). All the patients in two groups were males. IOFBs were successfully removed in 14 out of 15 patients in bar group. There were no significant differences between the two groups in IOFB location, mean size(length and width) of the foreign body, success rate of removing IOFB and the location of removing IOFB. The median time taken of removing FB was 295(265, 437.5) seconds (range 141–672 s) in forceps group, and 122(56.5,149.75) seconds (range 29–626 s) in bar group, with significantly difference(*P* = 0.001). None of the patients in bar group had IOFB slippage and fall, or related iatrogenic retinal damage in the process of removal. Slippage and fall during IOFB removal were recorded in 7 of 15(46.7%) in forceps group, significantly higher than bar group(*P* = 0.006). Iatrogenic retinal lesions during IOFB removal were recorded in 6 of 15(40.0%) in forceps group, significantly higher than bar group(*P* = 0.017). Iatrogenic retinal breaks during IOFB removal were encountered in 3 of 15(20.0%) in forceps group, two of which developed retinal detachment(13.3%). Detailed preoperative and intraoperative characteristics of the two approaches of removing IOFB were shown in Table 1. No endophthalmitis was found in patients in both groups during follow-up.

**Table 1:** Preoperative and intraoperative characteristics of the two approaches of removing IOFB

	Forcep group (*n* = 15)	Bar group (*n* = 15)	*P* value
Age, years(mean ± SD)	32.5 ± 8.2	36.2 ± 11.5	0.901^‡^
Male sex	15(100%)	15(100%)	**-**
Location of FB			
Vitreous	2	1	
Retina	13(86.7%)	14(93.3%)	0.82^†^
Size of FB(mm) (median, (Q1, Q3))(range)			
Length	2.2(1.75, 2.05) (1.5–3.2)	2.4(2, 2.8) (1.5–3.5)	0.39^#^
Width	1.9(1.6, 2.15) (1.2–3.1)	1.8(1.5, 2.3) (1.3–2.6)	0.98^#^
Success rate of removing FB(%)	15(100%)	14(93.3%)	> 0.99*
Location of removing FB			
Sclera	15(100%)	13(86.7%)	0.48*
Corneal limbus	0	2(13.3%)	
Time-taken to remove FB(seconds) (median,(Q1,Q3))(range)	295(265, 437.5) (141–672)	122(56.5,149.75) (29–626)	0.001^#^
Combined cataract extraction(%)	2(13.3%)	2(13.3%)	> 0.99*
IOFB slippage and fall (%)	7(46.7%)	0(0)	0.006*
Iatrogenic retinal injuries(%)	6(40.0%)	0(0)	0.017*
Iatrogenic retinal break(%)	3(20.0%)	0(0)	0.224*
Iatrogenic retinal detachment(%)	2(13.3%)	0	0.48*

IOFB, intraocular foreign body

* *P* value based on Fisher’s precise test

^#^*P* value based on Mann-Whitney U test

^†^*P* value based on chi-square test

‡*P* value based on independent t test

Note: The time taken to remove IOFB start to be from the moment the forceps or magnetic bar was inserted into vitreous cavity until the moment IOFB was completely removed

## Discussion

Removing foreign body from posterior segment of the eye is challenging. The purpose of the surgery is to remove the IOFB successfully with minimum extra damage to the eye. PPV is advocated in removing IOFB among vitreoretinal surgeons, as it would result in better anatomic and functional outcomes than traditional external magnet [8]. Two basic instruments have been reported applied to remove metallic IOFB in PPV, including intraocular forceps and intraocular rare earth magnet [9, 10].

Currently, the most commonly used instrument in removing IOFB in PPV is intraocular forceps, since it can grasp the foreign body regardless of its nature under direct vision. However, There are some issues that should not be ignored during the process of removing IOFB using forceps. A proportion of foreign bodies are difficult to hold tightly thus fall onto retina frequently, for instance those with large size, irregular shape or smooth surface. Secondly, forceps would cause oppression to retina in the process of grabbing those IOFBs located on retina. Thirdly, foreign bodies could be stuck in the scleral incision site and fall back to retina many times [11, 12]. Iatrogenic retinal contusions, iatrogenic retinal breaks, even rhegmatogenous retinal detachment may occur due to such uncontrollable slippage, especially when dealing with sharp foreign bodies. The most serious complication is iatrogenic macular injuries which would cause severe visual impairment [13]. To avoid these problems, some specialized instruments and techniques have been invented. There are a few customized intraocular forceps that can facilitate removing IOFBs and prevent slippage [11, 14]. However, most hospitals only have limited surgical instruments. It is reported that the use of perfluorocarbon liquid or sodium hyaluronate gel can also reduce the risk of falling, but it need extra surgical procedures and the protective effect only last a short period of time [13, 15].

Intraocular rare earth magnet has been introduced as an alternative instrument in removing IOFBs [16, 17]. The effectiveness and efficiency compared between intraocular rare earth magnet and forceps in PPV have not been studied before. Our self-assembled intraocular rare earth magnet is composed of a magnetic bar and a cylindrical magnet, both made of NdFeB. The 24 mm length is long enough to be close to almost any IOFB, from scleral incision cite. PPV together with wide-angle system allowed us to perform the whole process of attracting and removing IOFB under direct vision, avoiding additional damage to ocular tissues. There are three advantages of our self-assembled intraocular magnet compared with gripping forceps. Firstly, foreign bodies can adhere to the magnetic bar directly, avoiding applying pressure to retina, as well as preventing IOFB from slipping and dropping, thus reduce the possibility of iatrogenic retinal lesions. In the present study, over half of the patients in forceps group had one or more times of IOFB slippage and fall. 60%(6/15) of them had iatrogenic injuries. Iatrogenic retinal breaks were recorded in 3 of the 15 patients, and 2 of the 3 patients developed retinal detachment. While none of the patients in the bar group had IOFB slippage or fall, as well as related iatrogenic retinal injuries. Secondly, using our assembled intraocular magnet would shorten the operating time of removing IOFB, compared with using forceps. The median time of removing IOFB in bar group was 122 s, which was significantly shorter than the forceps group(295 s). Thirdly, the assembled magnetic bar simplified the operation, while manipulating intraocular forceps well requires more dexterity and study curve.

The size of the foreign body is one of the factors that should be considered in the removal strategies. According to previous literatures, intraocular magnet was recommended for removing small foreign bodies(< 1 mm) [5, 18]. In our study, the average size of IOFBs were 2.48 mm×1.9 mm in bar group, which belonged to median-size(1-3 mm). Among which three cases were over 3 mm in length(3 mm×3.5 mm, 2.5 mm×3.5 mm and 2.4 mm×3.1 mm). It is suggested that the assembled intraocular magnet is suitable for not only small-sized foreign bodies, but also median-sized or even large sized ones. It is worth-noting that only metallic foreign bodies with magnetism can be removed by intraocular rare earth magnet. There was one case in bar group failed to be attracted by the assembled intraocular rare earth magnet, so we used forceps instead. It was confirmed that the foreign body was composed of nonmagnetic metal after the operation. The success rate of removing IOFB was 93.3%(14/15) in the bar group, while all the IOFBs were removed successfully by forceps. According to previous reports, over 95% of metallic IOFBs are magnetic [3, 19]. We considered that the assembled intraocular magnet is useful in removing most metallic IOFBs.

There are some limitations of this study, for instance small sample size, lacking of randomization, and the time difference between the two groups. Further prospective studies with a larger number of patients are required to confirm these findings.

In summary, compared with common forceps, this assembled intraocular magnet can greatly reduce the possibility of IOFB slippage and fall, prevent related iatrogenic retinal damage, and shorten the operation time of removing IOFBs. Most metallic IOFBs can be removed with a high success rate and efficiency in this way. The assembled intraocular magnet can be an useful tool in the removal of metallic IOFBs in 25-G PPV.

## Data Availability

All data is available from the corresponding author on reasonable request.
